# The Role of the Renal Dopaminergic System and Oxidative Stress in the Pathogenesis of Hypertension

**DOI:** 10.3390/biomedicines9020139

**Published:** 2021-02-01

**Authors:** Waleed N. Qaddumi, Pedro A. Jose

**Affiliations:** 1 Columbian College of Arts & Sciences, The George Washington University, Washington, DC 20052, USA; waleedrahman@gwmail.gwu.edu; 2 Department of Medicine, Division of Renal Diseases & Hypertension, The George Washington University School of Medicine and Health Sciences, Washington, DC 20052, USA; 3 Department of Physiology/Pharmacology, Division of Renal Diseases & Hypertension, The George Washington University School of Medicine and Health Sciences, Washington, DC 20052, USA

**Keywords:** blood pressure, dopamine, dopamine receptor, dopaminergic system, hypertension, kidney, oxidative stress

## Abstract

The kidney is critical in the long-term regulation of blood pressure. Oxidative stress is one of the many factors that is accountable for the development of hypertension. The five dopamine receptor subtypes (D_1_R–D_5_R) have important roles in the regulation of blood pressure through several mechanisms, such as inhibition of oxidative stress. Dopamine receptors, including those expressed in the kidney, reduce oxidative stress by inhibiting the expression or action of receptors that increase oxidative stress. In addition, dopamine receptors stimulate the expression or action of receptors that decrease oxidative stress. This article examines the importance and relationship between the renal dopaminergic system and oxidative stress in the regulation of renal sodium handling and blood pressure. It discusses the current information on renal dopamine receptor-mediated antioxidative network, which includes the production of reactive oxygen species and abnormalities of renal dopamine receptors. Recognizing the mechanisms by which renal dopamine receptors regulate oxidative stress and their degree of influence on the pathogenesis of hypertension would further advance the understanding of the pathophysiology of hypertension.

## 1. Introduction

The development of hypertension is determined by various factors, including genetics, habits, and environment, such as traffic noise and air pollution [1,2,3,4,5]. Both indoor and outdoor exposure to fine particulate matter (PM_2.5_) is associated with hypertension in humans [6]. Long-term exposure of rats or mice to PM_2.5_ causes hypertension that is related to impairment of sodium excretion [7,8]. In utero exposure to PM_2.5_ also causes hypertension in the offspring [9]. The kidney is a key organ that is involved in the regulation of sodium homeostasis and control of blood pressure [10,11,12]. Sodium retention in hypertension is associated with the failure of signals to decrease renal sodium transport when sodium intake is greater than what is needed to maintain a normal sodium balance [10,11,12]. Normal sodium balance is achieved by proper interactions among several organs, including the kidney, brain, heart, liver, intestines, muscle, skin, and immune system [13,14,15,16,17,18]. One of the main factors that maintains a normal sodium balance is the renal-selective action of dopamine produced by the kidney [19,20,21,22,23,24,25,26]. This effect can be independent of renal nerves [22,23], but renal nerves can modulate the renal actions of dopamine [24]. The natriuretic effect of intrarenal dopamine may be more evident under conditions of a moderate increase in sodium intake/volume expansion [25,26,27,28] but not with marked volume expansion that may be seen with very high sodium intake [29]. The role of renal dopamine and sodium excretion can also be influenced by ingested nutrients, e.g., miso soup increases urinary dopamine production [30]. Fava bean seedling contains the precursor of dopamine, L-dihydroxyphenylalanine (L-DOPA), which increases renal dopamine production [31]. The increase in urinary dopamine is associated with an increase in sodium excretion [31]. By contrast, fava bean, which increases urinary dopamine and urinary norepinephrine, does not increase sodium excretion [32], probably because norepinephrine antagonizes the ability of dopamine to inhibit renal sodium transport [33]. It should be noted, however, that the L-DOPA content of fava bean is 1/10 that of fava bean seedlings [31]. Prolonged hydralazine therapy in patients with stable essential hypertension induces a defect in DOPA decarboxylation, which is needed to convert L-DOPA to dopamine, that is remediable by pyridoxine supplementation. Catechol-O-methyltransferase (COMT) which degrades dopamine, epinephrine, and norepinephrine to 3-methoxytyramine is inhibited by mercury and cadmium and causes hypertension, probably due to the increase in epinephrine and norepinephrine concentrations [34].

The circadian rhythm of sodium excretion (daytime > nighttime) has been suggested to be related to renal dopamine production [35]. Other variables that are important regarding the role of renal dopamine production and sodium excretion in humans include age [36,37,38,39], body mass [40], ethnicity/race [40,41,42,43], genetics [44], mineral intake [45,46], and sex [39,47]. Aging is associated with a decrease in urinary dopamine and its natriuretic effect [36,37,38,39]. The activity of the enzyme aromatic L-amino acid decarboxylase (AADC), which converts L-DOPA to dopamine, is greater in the kidneys of female than male mice [47]. Lean male, relative to lean female Zucker rats, have lower renal expression of two of the five dopamine receptor subtypes, D_1_R and D_3_R. Obese Zucker rats, relative to lean Zucker rats, have decreased renal expression of three of the five dopamine receptor subtypes, D_1_R, D_4_R, and D_5_R, in both male and female rats but D_3_R is increased in female rats [48]. Humans in the normal weight range with essential hypertension have increased urinary dopamine, whereas overweight subjects have decreased urinary dopamine. The natriuretic effect of intravenously infused dopamine is decreased in overweight patients with essential hypertension, relative to non-overweight patients [49]. 

In rodents, rat/mouse strain [50,51,52,53] and source [54] also need to be taken into consideration in renal dopamine function. The function of renal dopamine receptors is impaired in hypertension, due in part, to oxidative stress [55,56,57,58]. In this article, we review the relationship between oxidative stress and the intrarenal dopaminergic system in the regulation of blood pressure and the abnormalities involved in the development of hypertension.

## 2. Oxidative Stress and Hypertension

Oxidative stress occurs when there is an imbalance between the production of reactive oxygen species (ROS), and reactive nitrogen species and the antioxidant defense systems [55,59,60,61,62,63,64,65,66,67,68]. ROS are produced by cell organelles, including the mitochondria, peroxisomes, and endoplasmic reticulum, and consist of free radicals and non-radical derivatives. Free radicals are a class of oxygen atoms that contain unpaired electrons that include superoxide (O_2_^−^), hydroxyl radical (OH^−^), lipid peroxyl-radicals (LOO^−^), and alkoxy-radicals (LO^−^). Non-radicals include H_2_O_2_, peroxynitrite (ONOO^−^), hypochlorous acid (HOCl^−^), lipid hydroperoxide (LOOH), ozone (O_3_), singlet oxygen (^1^O_2_), and reactive carbonyls [55,59,60,61,62]. ROS are naturally generated from various bodily reactions, such as the reduced form of nicotinamide adenine dinucleotide phosphate (NADPH) oxidase, cyclooxygenases, xanthine oxidases, lipogenesis, iron-catalyzed Fenton reaction, and nitric oxide synthases (NOS) [55,56,57,58,59,60,61,62,63,64,65,66,67,68,69,70,71]. A major site for the intracellular production of ROS is from the process of mitochondrial respiration that occurs in all cells, including vascular and renal mesangial and tubular cells [55,59,60,61,62,63,64,65,66,67,68]. In the rat kidney, NADPH oxidase accounts for about half of ROS production, with the remaining half from mitochondria [69]. Normal generation of ROS is important in cellular signal transduction [59,60,61,62,63,70]. Levels of ROS are decreased by endogenous and exogenous antioxidants. Endogenously generated antioxidants include enzymatic and non-enzymatic antioxidants, which consist of metabolic and nutrient forms [59,60,61,62] (Table 1).

Oxidative stress is involved in the pathogenesis of high blood pressure, associated with impairment in sodium excretion [10,11,53,55,57,58,69,72,73,74,75]. A number of studies, both in humans and experimental animal models, have shown that unrestricted ROS production and/or impaired antioxidant mechanisms play a role in the development of hypertension [55,58,73,74,75,76,77,78]. In animal studies, it was confirmed with the use of specific ROS generating gene-knockout mice (e.g., gp91phox^−/−^) that inhibition of ROS production prevents or ameliorates the development of hypertension [79]. By contrast, germline deletion of SOD, which is expressed in the kidney, increases blood pressure [80]. The role of renal ROS production was proved by the increase in blood pressure with the renal-selective silencing of paraoxonase 2, DJ-1 (also known as Park 7), or sestrin2, which have antioxidant properties [81,82,83]. In the kidney, oxidative stress causes hypertension by promoting renal vasoconstriction and disrupting sodium homeostasis. However, it should be stated that the overall effect of ROS on renal sodium transport is very complex and cannot be fully determined due to the contrasting influence of ROS, which can increase or decrease renal sodium transport [52,72].

## 3. Renal Dopaminergic System

Dopamine, an endogenous catecholamine, is an important regulator of renal function and blood pressure [19,20,21,56,57,58,84,85,86,87]. In the kidney, dopamine is synthesized in the renal proximal tubule from the dopamine precursors, L-DOPA and tyrosine, which are taken up from the circulation [19,88,89,90,91]. L-DOPA is converted by AADC to dopamine [88,91]. Dopamine produced in renal proximal tubule cells can move across the basolateral and apical membranes and into the peritubular space and tubular lumen, respectively, to act on receptors present in most nephron segments [21,56,57,58,84,85,86,87]. Saline loading increases urinary dopamine, in part, by increasing the egress of dopamine into the tubular lumen, rather than into the interstitium [21,92]. Due to the lack of expression of dopamine β-hydroxylase in renal tubules, the synthesized dopamine is not metabolized into norepinephrine [93,94], which can otherwise increase renal sodium transport. However, dopamine is degraded in renal tissues both by deamination, via monoamine oxidase (MAO) to 3, 4-dihydroxyphenylacetic acid (DOPAC) [95,96], by methylation, via COMT to 3-methoxytyramine [96], and by renalase [97]. Renal dopamine is metabolized by MAO, predominantly in the proximal tubule while COMT metabolizes dopamine in more distal nephron segments [98]. Hormones, such as atrial natriuretic peptide, increase renal dopamine production, not only by increasing renal dopamine synthesis, but also by decreasing dopamine degradation via COMT [99]. Newly synthesized dopamine in the dog, rat, and human kidney is rapidly deaminated [100]. Moreover, as aforementioned, dopamine synthesized by renal proximal tubules, is preferentially secreted into the renal tubular lumen, and not secreted into the circulation [21,92,101,102,103], but there is spill-over of DOPA into the circulation with increased salt intake [90,104]. The normal circulating concentrations of dopamine (picomolar range) [26,105,106] are not sufficiently high enough for the activation of dopamine receptors, as the affinity of dopamine to its receptors is in the nanomolar range [107]. However, high nanomolar to low micromolar concentrations can be attained in dopamine-producing tissues (e.g., renal proximal tubule and jejunum) [106,108,109,110]. Intrarenal dopamine production is subject to adjustments made in response to dietary NaCl intake. Most studies have shown a correlation between urinary dopamine and sodium excretion; an increase in urinary dopamine is associated with an increase in urinary sodium excretion and a decrease in urinary dopamine is associated with a decrease in urinary sodium excretion [21,22,25,26,27,28,101,110,111,112,113]. However, this process is under genetic regulation [106]. In addition, age is considered as a factor in the amount of renal dopamine production, where relative to adults, dopamine synthesis is less in young and old humans and rodents [40,41,42,114,115,116,117]. In rodents, the age-related differences in renal dopamine synthesis may be strain-dependent [115,117]. In the brain, the amount of dopamine release is decreased by both D_1_-like and D_2_-like receptors [118,119,120]. However, in the kidney, the increase in renal dopamine production induced by uninephrectomy is further increased by a high salt intake [17,121].

The regulation of blood pressure by dopamine is different between the kidney and central nervous system. The increase in the activity of the renal dopaminergic system with the increase in the intake of salt prevents the development of hypertension [120,121]. The renal spill-over of dopamine into the circulation with salt loading [104] does not extend into the brain because dopamine does not cross the blood–brain barrier [122]. The delivery of dopamine-loaded poly(lactic-co-glycolic acid) nanoparticles into the brain that reached the striatum and substantia nigra of rats with Parkinson’s disease did not increase blood pressure [123]. It must be noted that the Parkinson’s disease in these rats was caused by 6-hydroxydopamine which destroys dopaminergic nerves. However, an overactivity of the dopaminergic system in certain regions in the brain, such as the amygdala and nucleus tractus solitarius, causes hypertension, but not in other brain regions such as the area postrema and locus coeruleus [103,124]. Rats made hypertensive by decreasing blood flow to one kidney have increased levels of dopamine and dopamine catabolites in the brain striatum [125]. Decreasing dopamine levels in the nigrostriatum of spontaneously hypertensive rats (SHRs) inhibits the development of hypertension [124]. However, monkeys made hypertensive by constricting the aorta have decreased D_1_-like receptor binding in the prefrontal cortex [126]. Additionally, SHRs have decreased postsynaptic dopaminergic and cholinergic functions in the ventrolateral striatum [127], reinforcing the similarities and differences on the regulation of blood pressure between the dopaminergic system inside and outside the central nervous system, such as the kidney. 

## 4. Impaired Dopamine Receptor Function and Hypertension 

**Figure 1:**
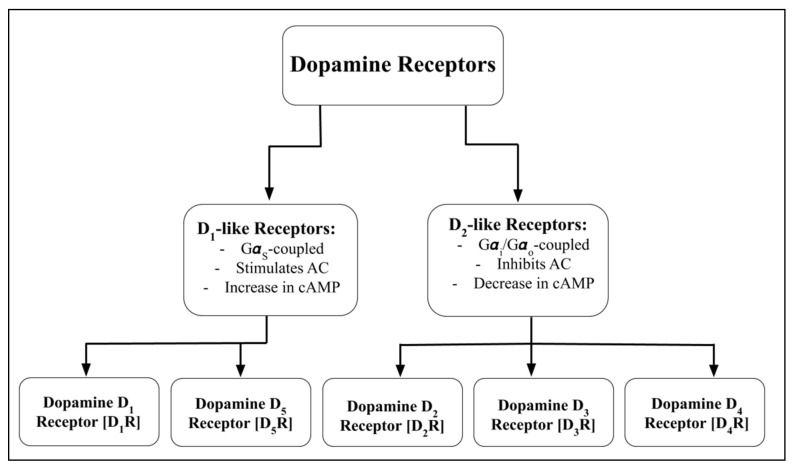
Schematic diagram summarizing dopamine receptor subtypes. G***α***_S_, G_S_ alpha subunit; G***α***_i_/G***α***_o_, G_i_ alpha subunit/G_o_ alpha subunit; AC, Adenylyl Cyclase; cAMP, Cyclic Adenosine Monophosphate.

Dopamine, via its five receptor subtypes, acts in an autocrine/paracrine manner to regulate renal tubular transport of sodium [120,121]. Dopamine receptors, belonging to the rhodopsin family (Class A) of seven-transmembrane G protein-coupled receptors (GPCRs), are classified into two families: D_1_-like receptors (dopamine D_1_ receptor [D_1_R] and dopamine D_5_ receptor [D_5_R]) couple to stimulatory G protein Gα_S_ and stimulate adenylate cyclase (AC) activity, whereas D_2_-like receptors (dopamine D_2_ receptor [D_2_R], dopamine D_3_ receptor [D_3_R], and dopamine D_4_ receptor [D_4_R]) couple to inhibitory G protein Gα_i_/Gα_o_ and inhibit AC activity [56,57,58,84,85,86,87,107,120,121] (Figure 1).

**Figure 2:**
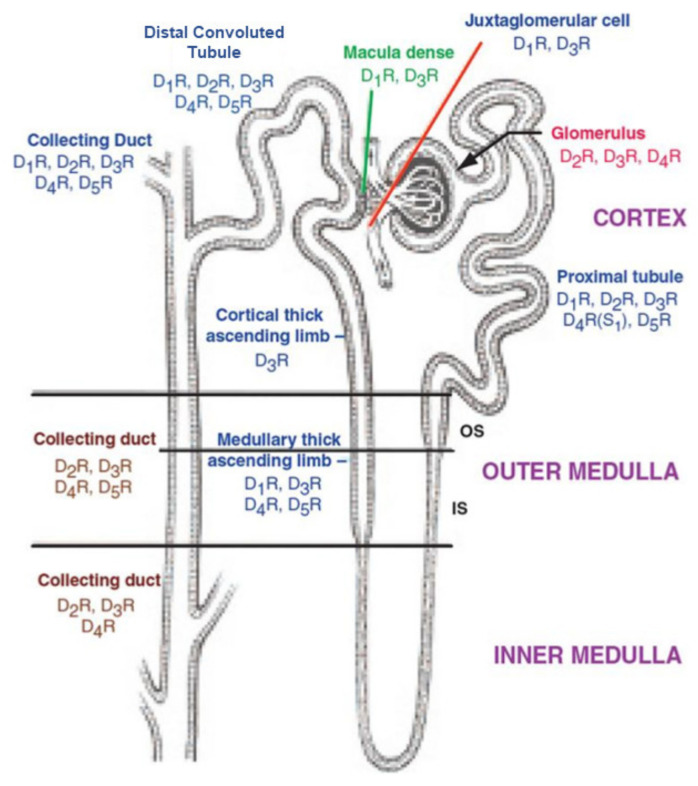
Diagram displaying the distribution of dopamine receptor subtypes (D_1_R-D_5_R) along the parts of a nephron. OS: outer stripe; IS: inner stripe; S_1_: first segment of the proximal tubule [121].

The expression of dopamine receptor subtypes in nephron segments varies among species [121]. All the five dopamine receptor subtypes are expressed in the proximal tubule, distal convoluted tubule, and cortical collecting duct (Figure 2).

The D_1_R and D_3_R are expressed in the macula densa and juxtaglomerular cell. The D_1_R, D_3_R, D_5_R, and maybe the D_4_R are expressed in the medullary thick ascending limb. Only the D_3_R is expressed in the cortical thick ascending limb. All the dopamine receptor subtypes are expressed in the distal convoluted tubule and cortical collecting duct. The D_2_R, D_3_R, D_4_R, and D_5_R are expressed in the outer medullary collecting duct, while only the D_2_R, D_3_R, and D_4_R are expressed in the inner medullary collecting duct. Rodent podocytes express the D_1_R and D_3_R but not D_2_R [128,129,130,131,132,133]. Mesangial cells express D_1_-like [131] and D_2_-like [132,133] receptors, but the exact subtypes have not been identified by reverse transcription-polymerase chain reaction (RT-PCR) or immunohistochemistry, using subtype-specific antibodies. In all studied species, there are no dopamine receptors in the thin descending and thin ascending limb of the nephron. However, dopamine has been reported to stimulate prostaglandin E2 production in primary cultures of the lower portion of the thin limb of Henle of rats [134].

Variants of human dopamine receptor subtype genes and their regulators are associated with hypertension [86,135,136,137,138]. Global disruption of any dopamine receptor gene in animal models results in high blood pressure, indicating the importance of dopamine receptors in the pathogenesis of hypertension that may be salt-sensitive [139,140,141,142,143,144,145]. The results, however, are not always consistent. The germline deletion of *Drd3* has been reported to increase blood pressure by two reports [142,143] but not by another report [146]. The reason for this discrepancy is not readily apparent; all the mice are in the same C57Bl/6 background (vide infra). The importance of the kidney in the regulation of blood pressure, as related to dopamine receptors, is attested by the normalization of the high blood pressure with the renal-selective rescue of the *Drd2* in mice with renal-selective silencing of *Drd2* [147]. The renal transplantation of a kidney from a *Drd5* knockout mouse, which is hypertensive, to a nephrectomized wild-type mouse, which is normotensive, promotes hypertension while the renal transplantation of a kidney from a wild-type mouse to a nephrectomized *Drd5* knockout mouse, which is hypertensive, normalizes blood pressure [148].

## 5. Renal Dopamine D_1_ Receptor [D_1_R], Oxidative Stress, and Hypertension

Dopamine regulates renal ion transport, in part, through the activation of the D_1_-like receptors [120,121]. In normotensive dogs and rats, the renal-selective stimulation of D_1_-like receptors, D_1_R and D_5_R, increases the excretion of sodium and other ions [23,120,121,149,150,151,152]; this effect is not seen in the SHR [153]. The D_1_R inhibits renal ion transport by direct inhibition of the sodium-hydrogen exchanger type 3 (NHE3) [154,155,156,157,158], sodium phosphate cotransporter type 2 (NaPi2) [159] NaHCO3 exchanger (NBCE1) [160,161,162], chloride bicarbonate (Cl^−^/HCO3^−^) exchanger (SLC26A6) [163], and Na^+^/K^+^-ATPase [164,165]. On a high NaCl diet, fenoldopam, a D_1_-like receptor agonist, causes natriuresis by inhibiting renal proximal and distal tubule sodium transport. By contrast, on a low NaCl diet, the increased renin-angiotensin activity prevents the D_1_-like receptor from inhibiting renal proximal tubule sodium transport, neutralizing the natriuretic effect of fenoldopam [166]. The D_1_-like receptor that mediates inhibition of distal nephron sodium transport has not been determined but this may be due to D_5_R rather than D_1_R. The expressions of the sodium-potassium-2 chloride cotransporter (NKCC2), sodium chloride cotransporter (NCC), and α and γ epithelial sodium channel (ENaC) are increased in D_5_R knockout mice [167]. 

The D_1_R also decreases renal ion transport by interacting with natriuretic hormones and receptors and antinatriuretic hormones and receptors. Thus, the D_1_R adds to the inhibitory effect on ion transport caused by natriuretic hormones, such as angiotensin 1–7 [168], atrial natriuretic peptide [169], and prolactin [170], and receptors such as the angiotensin II type 2 receptor (AT_2_R) [171], and gastrin/cholecystokinin B receptor (CCKBR) [172] but decreases the stimulatory effect of renal ion transport caused by angiotensin II (Ang II) [166,173] and α1-adrenergic receptor [33]. 

The D_1_R function in the kidney is also regulated by the location of its expression in cell membranes and compartments. In normotensive Wistar–Kyoto (WKY) rats, where D_1_R function is normal, D_1_R is found at the microvillous brush border and apical membranes [174,175]. However, in SHRs, where D_1_R function is impaired [176], it is found mostly in the cytosol [177]. Impaired D_1_-like receptor-mediated inhibition of sodium transport is also observed in humans with essential hypertension [178].

Dopamine has a biphasic effect on ROS production in human lymphocytes; low concentrations (≤5 μM) decrease, while high concentrations (≥100 μM) increase ROS production [179]. However, in opossum kidney cells, low concentrations of dopamine (nM) increases ROS production [180]. By contrast, in human renal proximal tubule cells, low and high concentrations of D_1_-like receptor agonists (e.g., fenoldopam) decrease ROS levels by decreasing ROS production and increasing ROS degradation [181,182]. D_1_R inhibits the activity of the pro-oxidant enzyme, NADPH oxidase (NOX), and NOX2 and NOX4 expressions in renal proximal tubule cells [181,182] and vitalizes the antioxidant enzyme paraoxonase 2 (PON2) to prevent oxidative stress [183]. D_1_-like receptors also decrease ROS production by increasing the expression of phase II antioxidant enzymes such as glutathione peroxidase, superoxide dismutase-1 (SOD-1), and glutamyl cysteine transferase that involves Nfr-2 [184]. Oxidative stress can also impair the ability of D_1_R to inhibit renal sodium absorption, resulting in a decrease in sodium excretion, and eventually causing hypertension [185,186,187,188]. Conditions, such as inflammation and hyperglycemia, can negatively affect the function of D_1_-like receptors, in part via D_1_R, by creating an imbalance between oxidant and antioxidant systems, where oxidant systems exceed antioxidant systems which results in oxidative stress [129,189]. However, the role of oxidative stress in the hypertension of D_1_R knockout mice has not been determined. Nevertheless, oxidative stress has the capability to suppress D_1_R gene transcription and signaling by stimulating activator protein 1 (AP-1) and specificity protein 3 (SP3) [190].

## 6. Renal Dopamine D_2_ Receptor [D_2_R], Oxidative Stress, and Hypertension

D_2_R, as with the D_1_R, has many effects on the kidney, including alleviating kidney injury and inflammation, inhibiting renal sodium transport, and maintaining normal blood pressure [121,147,191,192,193,194]. There are three isoforms of D_2_R: D_2_R-short, D_2_R-long, and D_2_R-longer [195,196,197]; the D_2_R-longer expression in the brain is only about 2–3% of the D_2_R-short and D_2_R-long [195]. The D_2_R-short inhibits adenylate cyclase activity to a greater extent than D_2_R-long. The D_2_R-short, exogenously expressed in human embryonic kidney cells, also increases dopamine transporter cell surface expression in human embryonic kidney cells [196]. The D_2_R-short is presumed to be an autoreceptor (presynaptic) while the D_2_R-long is postsynaptic [197]. The D_2_R controls the renal synthesis of dopamine [198,199], presumably by the D_2_R-long, which is the isoform expressed in the kidney [200].

In the renal cortical collecting duct, D_2_R (isoform not determined) inhibits basolateral K^+^ channels Kir4.1/5.1 and Kir4.1 channels [201] that can eventually affect NCC and chloride-channel protein Cl-Kb activities [202,203]. In the cortical collecting duct, the D_1_-like but not D_2_-like receptors can inhibit Na^+^/K^+^-ATPase activity [204]. In the rat renal proximal convoluted tubule, the dopamine-mediated inhibition of Na^+^/K^+^-ATPase activity is markedly attenuated [205] in the presence of D_1_-like receptor (SCH23390) [206] or D_2_R and D_3_R antagonist (S-sulpiride) [207]. The D_2_R probably negatively regulates NHE3 and NCC expressions because their renal expressions are increased in *Drd2^−/−^* mice [199]. Between the WKY rat and SHR, there are no noticeable differences in the expression and allocation of renal D_2_R other than that D_2_R is expressed in the glomeruli of WKY but not SHR [208]. 

**Figure 3:**
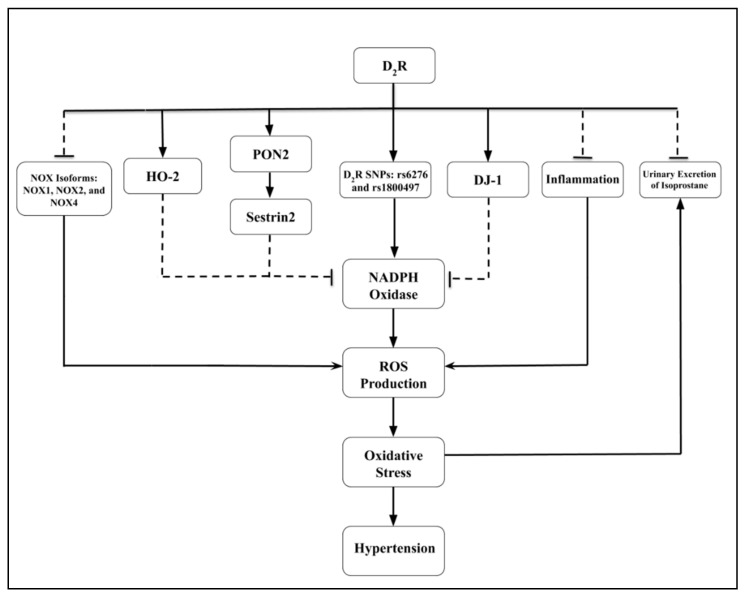
Schematic diagram displaying the role of renal D_2_R and interrelated components on the development of hypertension. The *dashed lines* illustrate inhibitory effects and *solid lines* illustrate stimulatory effects. D_2_R, Dopamine D_2_ Receptor; D_2_R SNPs, Dopamine D_2_ Receptor Single Nucleotide Polymorphisms; DJ-1, Park 7; HO-2, Heme-Oxygenase-2; NOX, NADPH oxidase; PON2, Paraoxonase 2.

The D_2_R is important in the regulation of blood pressure because germline deletion of *Drd2* in mice causes hypertension [140,141] that is salt-sensitive [141]. *Drd2* siRNA-renal-selective deletion of *Drd2* also increases blood pressure but salt sensitivity was not tested [147,193]. As with the D_1_R, the D_2_R regulates ROS production by inhibiting pro-oxidant systems and enabling antioxidant systems [199,209,210] (Figure 3). 

One of the mechanisms that allows D_2_R to regulate blood pressure is by decreasing oxidative stress in the kidney; germline deletion of *Drd2* in mice increases the renal activity of NADPH oxidase and expressions of NOX1, NOX2, and NOX4 and urinary excretion of isoprostane, a product of the non-enzymatic oxidation of arachidonic acid, and decreases the renal expression of the antioxidant enzymes, heme oxygenase 2 (HO-2), paraoxonase 2 (PON2), and sestrin2 but not heme oxygenase-1 (HO-1) [81,82,83,199,209,210,211,212]. Apocynin, a reduced NADPH oxidase inhibitor, or hemin, an inducer of HO-1, normalized the blood pressure of *Drd2^−/−^* mice [210]. It should be noted that the D_2_R in the striatum may actually increase ROS production [211]. However, renal ROS production is increased with the renal-selective silencing of *Drd2* which also increases blood pressure [193,194,199]. The stimulation of D_2_R in human renal proximal tubule cells decreases hyperoxidized peroxiredoxins and ROS production [83]. The antioxidant effect of D_2_R involves its interaction with proteins, including PON2, DJ-1, and sestrin2 [81,82,83]. PON2 inhibits NADPH oxidase activity, ROS production, and maintains blood pressure within the normal range. D_2_R interacts with both, PON2 and DJ-1, in human renal proximal tubule cells [81,82,83] (Figure 3). DJ-1, which is expressed in the mouse kidney, protects cells against harm that can be mediated by ROS [82,212]. Silencing DJ-1 in mice increased blood pressure, NADPH oxidase activity, uncoupling protein 2, and ROS production [82,212].

Sestrin2 is involved in augmenting the D_2_R effect to normalize blood pressure by decreasing ROS production and protecting against cellular damage [82]. At the same time, the stimulation of D_2_R increases the expression of sestrin2 [83] (Figure 3). 

In mice, the silencing of sestrin2 increased renal oxidative stress, inflammation, and blood pressure [83]. The expressions of the antioxidant proteins, PON2, sestrin2, and DJ-1, are increased by D_2_R stimulation and partially contribute to the inhibitory effects of D_2_R on ROS production [81,82,83]. There is an association between D_2_R-mediated inhibition of oxidative stress and inflammation; impaired D_2_R function would result in kidney damage and increased inflammation. Indeed, D_2_R single nucleotide polymorphisms (SNPs), such as rs6276 and rs1800497, decrease D_2_R expression and promote a proinflammatory and profibrotic phenotype in human renal proximal tubule cells [192,213] (Figure 3).

## 7. Renal Dopamine D_3_ Receptor [D_3_R], Oxidative Stress, and Hypertension

The D_3_R, as with the D_1_R and D_2_R, also maintains normal blood pressure, in part, by inhibition of renal ion transport [121], alleviation of kidney injury, and inhibition of inflammation, and ROS production [214]. There is tissue specificity of the beneficial effect of the D_3_R in the kidney because the D_3_R in astrocytes promotes inflammation [215]. Interestingly, the D_3_R is also anti-inflammatory in synovial mast cells [216] and mesolimbic neurons [217]. Renal-selective stimulation of D_3_R by the renal arterial infusion of PD128907 (D_3_R >> D_2_R) [218] or Z-1046 (D_3_R ≥ D_4_R > D_2_R) [219] increases sodium excretion in normotensive Wistar and WKY rats [218,219] but not in SHRs [219]. The D_3_R also interacts with the D_1_R [220], D_4_R [221], D_5_R [222], and endothelin B receptor (ETBR) [223] to inhibit Na^+^/K^+^-ATPase activity in rat renal proximal tubule cells from normotensive (WKY) but not hypertensive rats (i.e., SHR) [221]. The ability of dopamine to inhibit Na^+^/K^+^-ATPase activity in the rat proximal convoluted tubule [205] can be inhibited by YM 09151, a D_3_R and D_4_R antagonist [206], or sulpiride, a D_2_R and D_3_R antagonist [207]. The natriuresis caused by D_3_R stimulation in WKY rats is related to inhibition of Na^+^/K^+^-ATPase and NHE3 activities by interaction with Gα(12)/Gα(13) [224]. The disruption of *Drd3* in C57Bl/6 mice increases blood pressure [142,143] and decreases sodium excretion [142]. However, another study showed that the disruption of *Drd3*, also in C57Bl/6 mice, was not associated with an increase in blood pressure, regardless of the amount of sodium intake [146]. The reason for this discrepancy is not clear but as stated earlier, some differences in dopamine metabolism in the same species from different suppliers have been reported [54]. Although, the blood pressure is not increased in *Drd3^−/−^* mice in that one report [146], sodium excretion is lower in *Drd3^−/−^* mice than their wild-type controls [146]. These investigators also reported in a later study that the pharmacological blockade of D_3_R increases blood pressure in Dahl salt-resistant rats fed a high salt diet [225]. 

It is still not clear whether D_3_R has antioxidant effects in renal cells. The rat D_3_R heterologously overexpressed in HEK293 cells stimulates phospholipase D (PLD) activity [226]; a product of its enzymatic activity, phosphatidic acid, causes superoxide formation via NADPH oxidase [227]. However, as stated above, the D_3_R in mast cells in synovial fluid has antioxidant activity [216]. Moreover, D_3_R activation protects rat oligodendrocytes from free radical-mediated lipid peroxidation [228]. Pramipexole, a dopamine receptor agonist (D_3_R > D_2_R) [229], prevents the development of experimental autoimmune encephalomyelitis in mice [230]. Pramipexole has also a protective effect on H_2_O_2_-induced retinal damage in mice [231]. However, the neuroprotective effect of pramipexole may not be related to its antioxidant properties via D_3_R > D_2_R activation [232]. By contrast, the anti-inflammatory effect of PD128907 (D_3_R > D_2_R) in renal ischemia/reperfusion injury is associated with a decrease in ROS production [214]. However, hypertension associated with germline deletion of *Drd3* in mice is mild [142,143] and is not associated with oxidative stress [233]. This may be related to the increase in the renal expression of D_5_R which has antioxidant activities (vide infra).

## 8. Renal Dopamine D_4_ Receptor [D_4_R], Oxidative Stress, and Hypertension

The D_4_R, as with the D_1_R, D_2_R, and D_3_R, also maintains normal blood pressure, in part, by inhibition of renal ion transport [121,221]. Its role in inflammation in the kidney is not known but the D_4_R augments T helper 2 (Th2)-type allergic inflammation in the lung [234]. However, quinpirole (D_3_R = D_4_R > D_2_R agonist) attenuates the lymphocyte proliferation in response to concanavalin A (ConA) and decreases the IFN-γ but increases the interleukin-4 (IL-4) production [235]; IL-4 can be anti-inflammatory [236]. The role of D_4_R in renal oxidative stress is not known but activation of D_4_R protects against hypoxia/reoxygenation which increases intracellular ROS in a hippocampal neuronal cell line [237]. Clozapine, a drug with anti-D_4_R properties used for the treatment of schizophrenia, increases blood pressure [238]. Although renal Na^+^/K^+^-ATPase activity is not affected by germline deletion of *Drd4* in mice [144], the D_4_R agonist, PD168077 [239], inhibits Na^+^/K^+^-ATPase activity in WKY renal proximal tubule cells but in SHR renal proximal tubule cells [240]. The D_4_R also inhibits the expression of the insulin receptor and the ability of insulin to stimulate Na^+^/K^+^-ATPase activity in renal proximal tubule cells from WKY but not SHRs [241]. The D_4_R, as with the other dopamine receptor subtypes, participate in blood pressure regulation, by impairing the effect or expression of angiotensin II receptor type 1 (AT_1_R) [144,242]. The hypertension in *Drd4^−/−^* in mice is related in part to an increased AT_1_R activity; the expression of AT_1_R is increased in the organs studied, brain and kidney [144]. D_4_R also decreases AT_1_R expression in renal proximal tubule cells from WKY rats but increases it in renal proximal tubule cells from SHRs [242]. Conversely, angiotensin II increases D_4_R expression in renal proximal tubule cells from WKY and SHRs [240]. The D_4_R also mediates the dopamine-mediated inhibition of arginine vasopressin-dependent transepithelial sodium transport in the rat cortical collecting duct [243]. The presence of prejunctional D_4_R in the kidney is suggestive of its participation in neurotransmitter release in the kidney [244].

It is not clearly known if D_4_R has a direct antioxidative effect on the kidney. However, it can be surmised that D_4_R may have indirect antioxidant properties in the kidney. As stated above, the expression AT_1_R, which can increase ROS [245,246], is negatively regulated by D_4_R [144,242]. Furthermore, D_4_R does have antioxidative effects in neuronal and leukemic cells [247,248,249,250].

## 9. Renal Dopamine D_5_ Receptor [D_5_R], Oxidative Stress, and Hypertension

The D_5_R, as with the D_1_R, D_2_R, D_3_R, and D_4_R, also maintains normal blood pressure [121,145], in part, by inhibition of renal ion transport [165]. The D_5_R also interacts with the other dopamine receptors, D_1_R [165] and D_3_R [222], to inhibit Na^+^/K^+^-ATPase activity in renal proximal tubule cells from normotensive humans [165] and normotensive (WKY) rats [222]. This effect is impaired in the SHR [222]. As with the D_1_R, the D_5_R also decreases renal ion transport by adding to or enhancing the natriuretic effect of hormones and receptors, such as gastrin/CCKBR [251] and antagonizing the effect of antinatriuretic hormones and receptors, such as AT_1_R [252,253,254] and α-adrenergic receptors [33]. The D_1_R and D_5_R interact to inhibit NHE3 and Na^+^/K^+^-ATPase activity in human renal proximal tubule cells via the phospholipase C pathway [165]. The D_2_R, D_3_R, D_4_R, and D_5_R may regulate NCC because its expression is increased in *Drd2^−/−^* [199], *Drd3^−/−^* [255], *Drd4^−/−^* [256], and *Drd5^−/−^* [167] mice. The D_5_R may also regulate ENaC because α and γ subunit expressions are increased in *Drd5^−/−^* mice [167]. The hypertension in *Drd5^−/−^* mice is salt-sensitive [167], as is the case in *Drd2^−/−^* mice [141]. 

**Figure 4:**
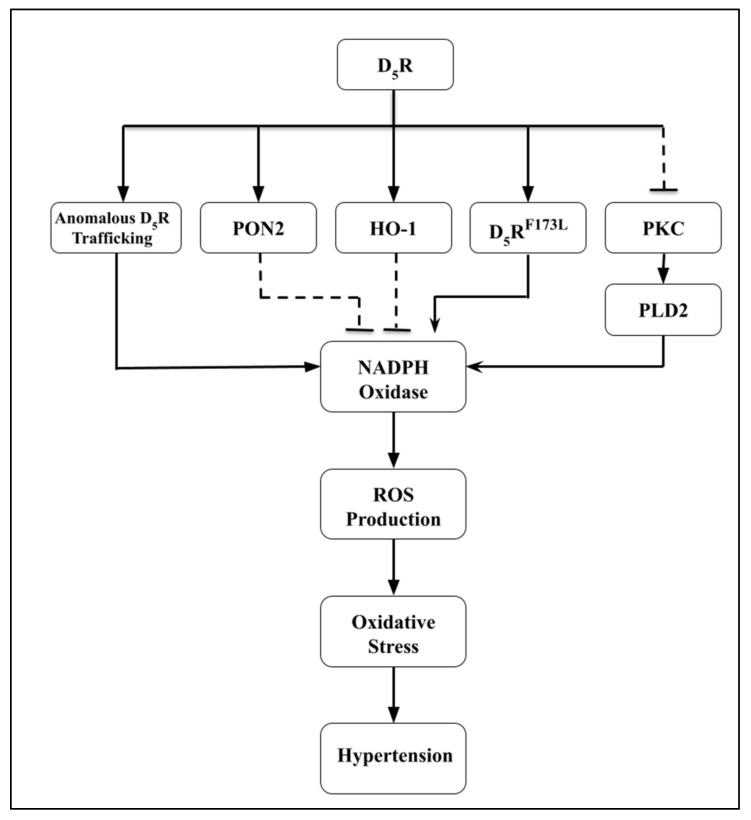
Schematic diagram displaying the role of renal D_5_R and interrelated components on the development of hypertension. The *dashed lines* illustrate inhibitory effects and *solid lines* illustrate stimulatory effects. D_5_R, Dopamine D_5_ Receptor; PON2, Paraoxonase 2; HO-1, Heme Oxygenase-1; PLD2, Phospholipase D2; PKC, Protein Kinase C.

Compared with the D_1_R, the D_5_R has a 10-fold higher affinity for dopamine and has trafficking features related to the third intracellular loop that is required for D_5_R endocytosis mediated by protein kinase C (PKC) [257,258,259]. As with the D_1_R and D_2_R, the antioxidant effect of D_5_R is related to the inhibition of NADPH oxidase expression and activity (Figure 4) [260,261,262]. The ability for D_5_R to inhibit NADPH oxidase activity may be related to the inhibition of PLD by D_5_R. PLD increases ROS synthesis; PLD2 but not PLD1 expression and activity are decreased when D_5_R is activated by the D_1_-like receptor agonist, fenoldopam, in HEK-293 cells heterologously expressing the D_5_R (HEK-hD_5_R) [263].

The D_5_R decreases ROS production not only by inhibiting pro-oxidant enzymes, such as NADPH oxidase but also by stimulating the activity of antioxidant enzymes. As is the case for the D_1_R [183] and D_2_R [81], the antioxidant enzyme PON2 participates in the D_5_R-mediated inhibition of ROS production [183]. The silencing of *DRD5* in human renal proximal tubule cells decreases PON2 expression and increases ROS production [183]. NADPH oxidase activity is decreased in HEK-hD_5_R cells expressing *HMOX1,* the gene product of which is HO-1, an antioxidant enzyme [260] (Figure 4). Thus, the increase in blood pressure and ROS production in D_5_R deficiency is related to the decrease in HO-1 and PON2 expression/activity. Certain *DRD5* SNPs hinder D_5_R function and sustain oxidative stress in the hypertensive state. Specifically, the human D_5_R173F > L (*hD_5_R^173F > L^*) mutation impedes cAMP production, increases renal NADPH activity, and increases AT_1_R expression which aids in the pathogenesis of salt-sensitive blood hypertension [252,262,264]. A pivotal factor in the impairment of D_5_R function and increased blood pressure is the hyperphosphorylation of *hD_5_^R173F > L^* [264] (Figure 4). Inflammation increases ROS production and vice versa [55]. The D_5_R has a complex effect on inflammation. The early inflammation in autoimmune experimental encephalomyelitis is potentiated by D_5_R signaling in CD4^+^ T cells but the D_5_R augments the anti-inflammatory effect of T regs [265]. The D_5_R also inhibits TLR2-induced NF-κB activation and inflammation in macrophages [266] and IFN-gamma production in natural killer cells [267]. The effect of the D_5_R in the inflammatory process in the kidney has not been determined.

## 10. Renal Dopaminergic and Renin-Angiotensin Systems Interaction in Oxidative Stress and Inflammation

As aforementioned, dopamine and the renin-angiotensin system interact in the kidney in the regulation of sodium transport. In general, whereas all five dopamine receptor subtypes inhibit sodium transport [120,121], the AT_1_R increases [252,253,254], while the AT_2_R decreases sodium transport [171]. D_1_-like receptors interact with angiotensin-(1–7) to inhibit renal tubular Na^+^/K^+^-ATPase and NHE3 activities [168]. In this situation, angiotensin-(1–7) increases dopamine production that is not related to receptor/receptor interaction. Decreasing renal dopamine production in mice allows unrestrained angiotensin II effects to increase renal sodium transport that is related to an increase in renal expression of AT_1b_R and decrease in AT_2_R and the angiotensin-(1–7) receptors (Mas) [19]. The AT_1_R increases ROS production [245,246,247], whereas the dopamine receptors decrease ROS production [52]. As stated above, inflammation increases ROS production and vice versa [55]; dopamine receptors [52] and AT_2_R [268] also decrease inflammation while the AT_1_R increases inflammation [245,246,247]. Angiotensin-(1–9) can decrease inflammation independent of the AT_2_R [269]. Thus, dopamine receptors and AT_1_R attenuate each other’s function while dopamine receptors and AT_2_R and Mas receptors may augment each other’s function.

## 11. Conclusions

Based on current evidence, the five dopamine receptor subtypes, D_1_R, D_2_R, D_3_R, D_4_R, and D_5_R, by themselves, by their interaction among themselves and with other genes, regulate renal tubular ion transport and ROS production. Dysfunction of any of the dopamine receptor subtypes impairs the ability to excrete a sodium load and decrease ROS production, eventually resulting in the development of hypertension. There are still elements that remain to be resolved and should be considered in future studies, including the antioxidant activity of D_3_R and D_4_R in the kidney. It has to be borne in mind that the effects of dopamine receptor subtypes on the regulation of sodium transport and ROS in renal cells may be different from that seen in other cells [180,211,216,226,234,265,270,271]. A better understanding of the relationship between renal dopamine receptors and oxidative stress in the regulation of renal tubular function and blood pressure would improve our view on the pathogenesis and treatment of hypertension.

## Figures and Tables

**Table 1:** Table classifying the different forms of endogenous antioxidants with appropriate examples.

Endogenous Antioxidants		
Enzymatic Antioxidants	Non-Enzymatic Antioxidants	
Antioxidants	Metabolic Antioxidants	Nutrient Antioxidants
Arylesterase Catalase Coenzyme Q_10_ (ubiquinol) Glutathione-dependent enzymes ○ Glutathione Peroxidase ○ Glutathione Reductase ○ Glutathione S-transferase Heme Oxygenase Paraoxonase-1 Peroxiredoxins Superoxide dismutase	Bilirubin Glutathione L-arginine Melatonin Quinones Thioredoxin Uric acid	Carotenoids Flavonoids Lipoic acid Polyphenols Polyunsaturated Fatty Acids Vitamin A Vitamin C Vitamin E (α-Tocopherol) Vitamin K_1_ (Ubiquinone)
